# Effect of Water Film Induced by Wet Shot Peening on Dimple Size and Residual Stress Distribution

**DOI:** 10.3390/ma18184347

**Published:** 2025-09-17

**Authors:** Chao Fang, Zhongjin Wang

**Affiliations:** 1 School of Materials Science and Engineering, Harbin Institute of Technology, Harbin 150001, China; 2 National Key Laboratory for Precision Hot Forming, Harbin Institute of Technology, Harbin 150001, China

**Keywords:** wet shot peening, finite element, water film, dimple, residual stress

## Abstract

Recently, considerable research has been conducted on wet shot peening (WSP), but a detailed investigation of this process is still lacking. For a systematic study, four three-dimensional models of WSP and shot peening (SP) were developed using the finite element method (FEM), based on the coupled Eulerian–Lagrangian (CEL) method. Micron-scaled water film is directly observed during WSP processing. Simulation results indicate that the water film has a significant impact on the dimple size and residual stress distribution. Compared with SP, WSP can produce (a) a dimple with a larger curvature radius, (b) greater compressive residual stress in the surface layer with a larger area, and (c) more uniformly distributed surface residual stress. This work reveals the mechanism underlying the changes mentioned above, which provides rationales for the promotional applications of WSP.

## 1. Introduction

The surface structures and properties of a material have significant impacts on its material properties, such as fatigue, friction, wear, and corrosion [1,2]. Over the past two decades, considerable attention has been devoted to shot peening (SP) processing, as it can significantly enhance material performance by inducing a fine grain or nanograin structure in the surface layer [3,4,5,6]. In addition to microstructure, surface roughness and residual stress, which are the byproducts of SP, also have an important influence on the material’s service performance.

In recent years, a number of SP-derived processes, such as pre-mixed water-jet peening [7] and post-mixed water-jet peening [8], have been developed. This article refers to pre-mixed water-jet peening as wet shot peening (WSP). WSP, as opposed to traditional SP, eliminates dust pollution, reduces noise pollution, obtains a more stable result, and achieves higher surface integrity, which attracts a lot of attention [9,10]. Unfortunately, there has been a paucity of studies on the mechanism underlying why WSP can achieve higher surface integrity.

Compared with experimental techniques, the finite element method (FEM) is much cheaper, more time-saving, and more efficient. For these reasons, the FEM has been widely used for SP. In 1971, Hardy et al. utilized the FEM to study the deformation and stress generated by a rigid ball pressed into an elastoplastic half-space in a two-dimensional scale, which was the earliest report on the use of the FEM to study SP [11]. In 1993, Kral et al. used ABAQUS to study the elastic–plastic contact of steel balls with a uniform half-space [12]. Following that, numerous studies have been conducted on shot peening modeling, process parameters, surface roughness, microstructure evolution, and stress–strain distribution [13,14,15,16,17,18]. FEM studies for WSP can be divided into two stages. In the first stage, the effect of water is neglected, and WSP is treated as SP [19]. In the second stage, the effect of water starts to be noticed for a more accurate result [20,21]. Zhong et al. investigated the surface roughness and residual stress field based on the coupled Eulerian–Lagrangian (CEL) method and validated them by experimental results [20]. Ren et al. researched the velocity variation with time during WSP using the smoothed particle hydrodynamics (SPH) method [21]. In the case of high strain, the CEL method is usually more accurate than the SPH method [22], which is employed only when the traditional finite element method or CEL method has reached its inherent limitations or is too expensive to implement. To find the water film and quantify its thickness, Dong et al. poured water on the surface of a 2A12 aluminum alloy plate. The average thickness of the water film was 1.138 mm, measured by modified vernier calipers [23]. The water film observed by Dong et al. was a static water film due to the surface tension of water and could not be applied to the dynamic WSP process. During WSP, a mixture of shot and liquid media impacts the target material at tens of meters per second, making it difficult to observe the water film and measure its thickness on site. In this case, the FEM may be a more appropriate choice for investigation. Zhou et al. studied the mechanical removal mechanism of silicon carbide substrate in a water-lubricated environment by molecular dynamics (MD). They found an Ångstrom-scaled water film between the shot and substrate [24]. Although the model was at a nanometer scale, which was different from the millimeter-scale model of WSP, at least it proves the existence of water film. However, experimental or simulation-based studies directly examining the dynamic evolution of the water film during WSP are still lacking. Compared with the nano-scale model developed by Zhou et al., the WSP model is too huge to use the MD method because of the high computational cost. Based on the above analysis, the CEL method is applied in this work. Many studies mentioned that WSP can form a buffer “water film” between target and shot, which accounts for the difference in surface integrity between WSP and SP. Yong et al. investigated the water-mixture film formed by water and powder particles such as steel powder and garnet abrasives. Their findings indicated that the film functions as a buffer and substantially modifies the impact dynamics between the shot and the target surface. Theoretical analysis further indicates that at low impact energies, the water film buffers the impact force. As the impact energy increases, the buffering effect stabilizes [25]. Feldmann applied WSP and SP with 200% coverage using ceramic shot to Ti6243 target materials. Surface roughness increased by 0.11 μm (Ra) after WSP and by 0.4 μm (Ra) after SP, demonstrating that wet shot peening produces a smoother surface [26]. Similarly, Yang conducted related research [27]. Feldmann also reported stress relaxation on the surface of shot-peened material after 200% coverage, while no stress relaxation was observed in wet shot-peened samples. These findings indicated that WSP enhances stress distribution stability due to the buffering effect of the water medium, whereas SP at high coverage induces stress relaxation through dislocation slip from concentrated energy. Furthermore, the application of WSP has been shown to improve material properties such as hardness, wear resistance, corrosion resistance, and fatigue resistance [28,29,30]. Despite this, detailed research on the water-film effect remains limited.

Comparative studies are used to systematically investigate the differences between WSP and SP. This work presents the first comprehensive modeling of the water-film effect using the CEL method during WSP. The effects of velocity and viscosity during WSP are also explored. Additionally, the reasons that WSP can obtain a larger curvature radius dimple and better residual stress distribution than those obtained by SP for a similar dimple depth are discussed, which provide theoretical supports for the broader application of WSP.

## 2. Finite Element Model

### 2.1. CEL Method

**Figure 1:**
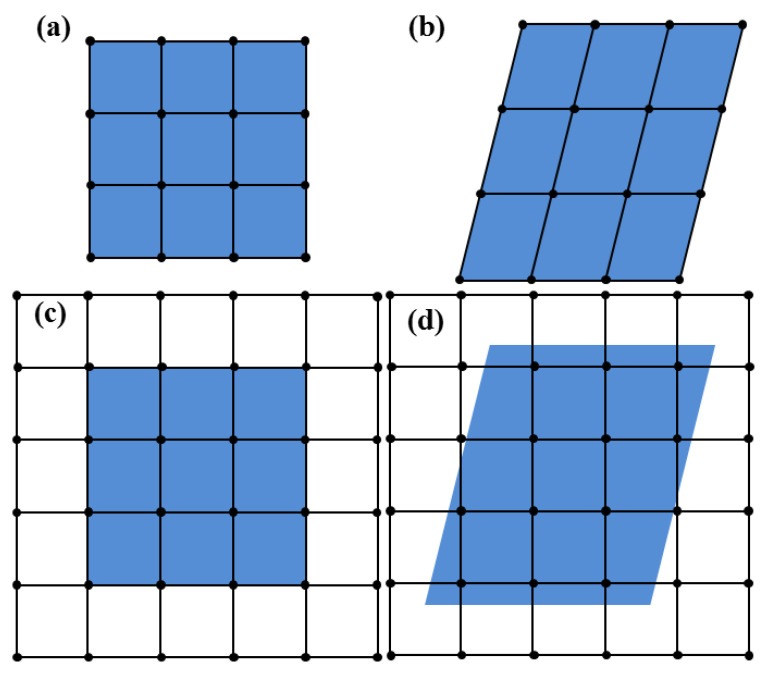
Illustration of deformations for Lagrangian analysis and Eulerian analysis: (**a**) original Lagrangian mesh, (**b**) deformed Lagrangian mesh, (**c**) original Eulerian mesh, (**d**) deformed Eulerian mesh. The blue area denotes the material.

Lagrangian analysis and Eulerian analysis are two basic techniques used in finite element analysis. For Lagrangian analysis, the material deforms with mesh deformation, and the element is filled with material, which is suitable for solid material. For Eulerian analysis, a mesh is completely fixed in space, and the material flows across elements, making it appropriate for fluid material. Figure 1 illustrates deformations for Lagrangian analysis (Figure 1a,b) and Eulerian analysis (Figure 1c,d).

**Figure 2:**
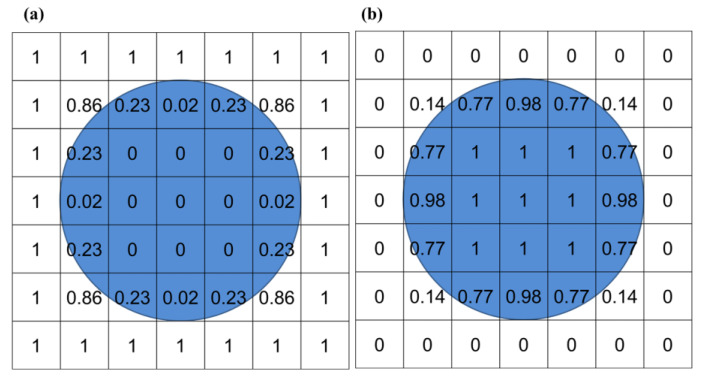
Concept of EVF in Eulerian analysis: (**a**) occupied volume assigned an EVF value of 0 (empty), (**b**) occupied volume assigned an EVF value of 1 (filled). The blue area denotes the material-filled region where EVF = 1.

The assignment of material within the Eulerian element is achieved by the Eulerian volume fraction (EVF). EVF comes from the Boolean comparison between a Eulerian part instance and the reference part instance. The value of EVF ranges from 0 to 1, which depends on the occupied volume fraction between two instances. The volume fraction tool in ABAQUS allows operators to choose whether the occupied volume is assigned a value of 1 or 0 (Figure 2) [22]. In this work, the occupied volume is assigned with 1 (Figure 2b). The CEL method uses general contact based on the penalty function method to handle the contact between Lagrangian material and Eulerian material. It has the advantages of both Lagrangian analysis and Eulerian analysis, which can realize solid–liquid coupling precisely.

### 2.2. Geometrical Model

**Figure 3:**
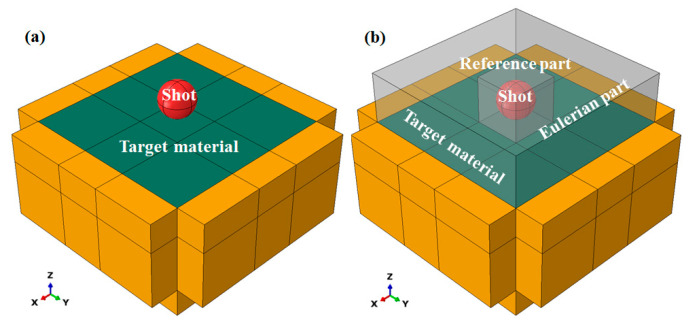
Geometrical models for (**a**) SP and (**b**) WSP. The shot is depicted in red, the target material in dark green, the infinite cells in yellow, and the Eulerian or reference part in gray.

Since industrial WSP involves multi-shot impacts with random angles/distributions, a full three-dimensional analysis is necessary. To align with subsequent studies on three-dimensional random WSP, a full three-dimensional model was used in this analysis. This work aims to investigate the difference between WSP and SP. The influences of parameters (such as impact angle, coverage, and surface roughness) are not considered. Based on the above reasons, simplified single-shot models are adopted. A three-dimensional WSP model and SP models are developed by ABAQUS/Explicit 2016 (Figure 3). The only distinctions between the WSP model and the SP models are that the WSP model has a Eulerian part instance and a reference part instance. The target (green part in Figure 3) is a 3 × 3 × 1.5 mm^3^ cuboid. The 0.8 × 0.8 × 0.5 mm^3^ impact area is located at the center of the target material. Five cuboids with a thickness of 0.5 mm are set around the target material except for the top position, and are used to assign infinite elements to eliminate the effect of elastic shear wave reflections. The shot diameter is 0.6 mm (red part in Figure 3), and the shot center is 0.4 mm from the upper surface of the target. The reference part instance (the smaller translucent cube in Figure 3b) is created by Boolean operation between a cube with a side length of 0.8 mm and the shot. The spatial coordinates of the cube center and shot center are the same. The Eulerian instance is a 3 × 3 × 1 mm^3^ cuboid (the bigger translucent cube in Figure 3b). In order to detect the water film between dimple and shot, the dimple should be located in the overlapping space between the Euler and the target material. The length of overlapped space in the *Z*-axis direction is set to 0.035 mm.

**Figure 4:**
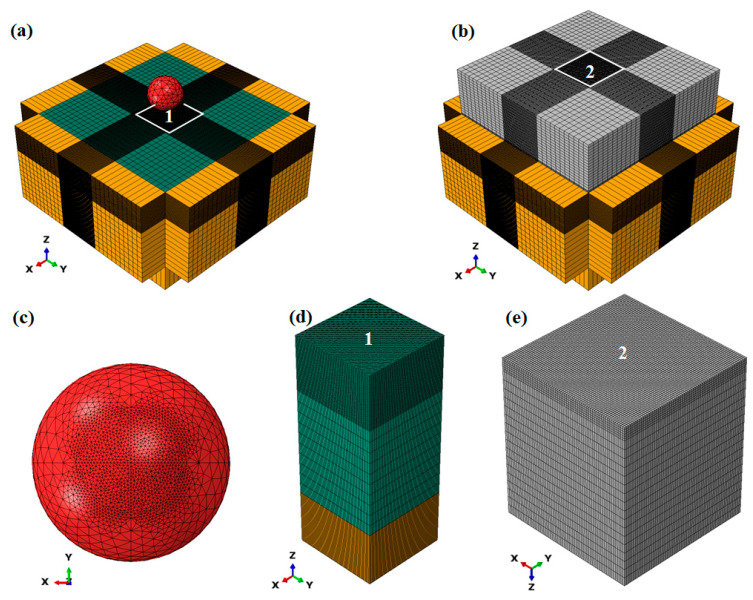
Element assignment for the models: (**a**) SP model, (**b**) WSP model, (**c**) close-up of mesh refinement in shot, (**d**) close-up of mesh refinement in region 1 of the (**a**) subfigure, (**e**) close-up of mesh refinement in region 2 of the (**b**) subfigure. Each color has the same meaning as described in the caption of Figure 3.

The details of the element assignment for the models are shown in Figure 4. Considering the computing accuracy and stability, the element size should be no larger than 1/15 of the dimple diameter [31]. The impact area of the shot, the impact area of the target, and the Eulerian area near the impact area are meshed with a size of 10 μm, whichis less than 1/15 of the 154.99 μm minimum dimple size (see Section 3.3). To achieve high computational efficiency, the element sizes in other areas of the models are coarsened appropriately based on 10 μm. Modified three-dimensional ten-node quadratic tetrahedral elements (C3D10M) are used for shots with 43,625 nodes and 30,270 elements. Because the elastic modulus of the ceramic shot is approximately three times greater than that of the Ti6Al4V target (Table 1), deformation of the shot during impact can be considered negligible. Furthermore, the internal stress distribution within the shots is not of concern. The shots are therefore modeled as rigid bodies, which increases computational efficiency. The shots are set as rigid bodies with the reference points at the shot centers using the rigid body constraint in ABAQUS, which requires that the rigid bodies be meshed with material properties. Three-dimensional eight-node linear reduced integration elements (C3D8R) are applied for targets with 898,576 nodes and 870,016 elements. The three-dimensional eight-node linear reduced integration Eulerian elements (EC3D8R) are applied for Eulerian instance with 194,400 nodes and 182,183 elements.

Generally, the velocity of SP ranges from 20 m/s to 100 m/s [32]. This work set the initial shot velocity as 50 m/s for WSP (WSP-50 m/s). For comparative studies, three different SP models are developed with the initial velocities of 42.88 m/s (SP-42.88 m/s), 46 m/s (SP-46 m/s), and 50 m/s (SP-50 m/s). The velocities are selected to enable three targeted comparisons: (a) Energy-based: SP-42.88 m/s matches the effective impact velocity (shot velocity at the beginning of the impact) of WSP-50 m/s (see Section 3.2; (b) Geometry-based: SP-46 m/s yields dimples equivalent in size to WSP-50 m/s; (c) Process-based: SP-50 m/s has the same initial velocity as WSP-50 m/s. The initial state of the water in WSP-50 m/s is achieved by EVF with a velocity of 50 m/s. The impact angles for both WSP and SP are 90°.

Surface-to-surface contact is applied for the interaction between shot and target. The master surface is the shot surface, and the slave surface is the upper surface of the target. A penalty algorithm is used with a friction coefficient of 0.2 [33]. The criterion for the shot–water interaction and target–water interaction is general contact with a friction coefficient of 0.02 [34]. The lower surface of the target is fixed (U1 = U2 = U3 = UR1 = UR2 = UR3 = 0). For modeling, the large deformation option (Nlgeom) is selected.

### 2.3. Material Model

#### 2.3.1. Johnson–Cook Constitutive Law

In this work, the target and shot are made of Ti6Al4V and ceramic, respectively. Their elasticity modules, densities, and Poisson’s ratios are shown in Table 1 [20,35].

Strain rate and strain hardening are taken into account by the Johnson–Cook constitutive law, which is suitable for transient metallic material deformation with a high strain rate. The law is used for Ti6Al4V with the following form:

(1) $$\bar{\sigma }\;=\;\left[A\;+\;B{\left({\bar{\varepsilon }}^{pl}\right)}^{n}\right]\left[1\;+\;C\;\ln\left(\frac{{\overset{•}{\bar{\varepsilon }}}^{pl}}{\overset{•}{\varepsilon_{0}}}\right)\right]\left(1-{\hat{T}}^{m}\right)$$

With

(2) $${\hat{T}}^{m}=\frac{T-T_{\mathrm{room}}}{T_{\mathrm{melt}}-T_{\mathrm{room}}}$$

where $\bar{\sigma }$ is flow stress, ${\bar{\varepsilon }}^{pl}$ is equivalent plastic strain, ${\overset{•}{\bar{\varepsilon }}}^{pl}$ is equivalent plastic strain rate, $\overset{•}{\varepsilon_{0}}$ is reference strain rate, A is yield stress, B is strain hardening constant, *n* is strain hardening exponent, C is viscous effect constant, m is heat softening index, T_melt_ is melting point, T_room_ is room temperature. The above parameters of Ti6Al4V are listed in Table 2 [35].

#### 2.3.2. Mie–Grüneisen Equations of State

The Mie–Grüneisen equation of state defines the relationship between liquid pressure, density, and internal energy. It can be used to describe the behavior of water during WSP. The linear U_s_ − U_p_ form of the Mie–Grüneisen equation of state is

(3) $$p=\frac{\rho_{0}c_{0}^{2}\eta }{{\left(1-s\eta \right)}^{2}}(1-\frac{\Gamma_{0}\eta }{2})+\Gamma_{0}\rho_{0}Em$$

With

*η* = 1 − *ρ*_0_/*ρ* (4)

U_s_ = *c*_0_ + s·U_p_ (5)

where *p* is pressure, $\rho_{0}c_{0}^{2}$ is equivalent to the elastic bulk modulus at small nominal strains, *ρ*_0_ is reference density, *ρ* is density, *c*_0_ is the fluid velocity of sound, *η* is nominal compressive strain, Γ_0_ is material constant, *E_m_* is internal energy per unit mass, U_s_ is shock velocity, U_p_ is particle velocity, *s* is the slope of U_s_ − U_p_ equation. The parameters of water are provided in Table 3 [36].

### 2.4. Validation of the Finite Element Model

**Figure 5:**
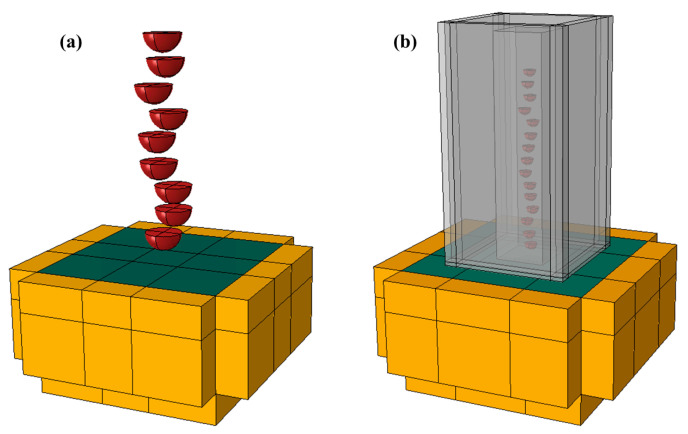
Validation models for (**a**) SP and (**b**) WSP. Each color has the same meaning as described in the caption of Figure 3.

The finite element models were validated using experimental data reported in the literature [37,38]. The SP target material was Ti6Al4V, and the shot material was cast steel. Process parameters included an Almen intensity of 0.3 mmA, 100% coverage, air pressure of 3 bar (air pressure), shot flux of 1.5 kg/min, and shot diameter of 0.6 mm. The velocity was 50 m/s, calculated as described in the following formula [39]:

(6) $$\;v=\;\frac{16.35\times p}{1.53\times m+p}\;+\;\frac{29.50\times P}{0.598\times d+p}+4.83\times P$$

where *p*, *m*, *d* represent the jet pressure (bar), the flux of shot balls (kg/min), and the diameter of shot balls (mm), respectively. To reduce computational cost, the shots were modeled as a hemisphere with a material density set to twice that of the raw material. A random SP model was constructed for the 0.3 × 0.3 mm^2^ collision region, as shown in Figure 5a.

The WSP utilized Ti6Al4V as the target material and ceramic as the shot material. The Almen intensity was 0.3 mmA, and the shot diameter was 0.2 mm. The effective impact velocity was 52 m/s, as determined by the following formula [40]:

(7) $$A_{H,\;ceramic}\left(D,v\right)=0.05714D+0.01019Dv-0.00002472Dv^{2}-0.00003583D^{2}v^{2}$$

where A_H,ceramic_ (D, v), D, v represent the Almen intensity, shot diameter, and effective impact velocity of the shot, respectively. A_H,ceramic_ (D, v) is expressed in mmA, D is expressed in mm, and v is expressed in m/s. During WSP, the shot velocity is affected by the liquid before impacting the target, resulting in a difference between the initial velocity and effective velocity. After several finite element simulation tests, the initial velocity was obtained as 68 m/s. The shots were modeled as hemispheres with double the original density, consistent with the approach used in SP. A random WSP model was constructed for the 0.1 × 0.1 mm^2^ collision region, as illustrated in Figure 5b.

**Figure 6:**
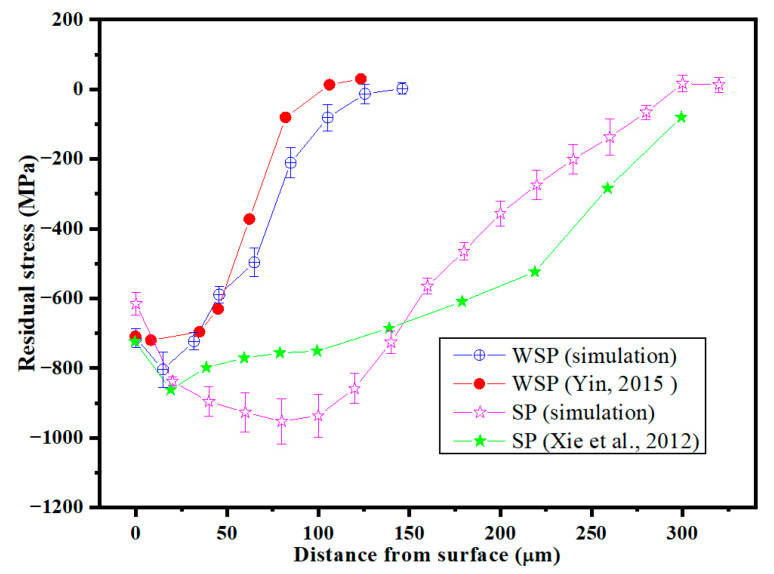
Comparison between the predicted residual stresses and experimental results [37,38].

Following the simulation, five points were taken at each different depth of the target, and the average residual stress at each depth was calculated. Figure 6 presents both the simulation data and the corresponding experimental results. Despite variations in the specific values, the overall patterns and trends observed remain consistent across the data sets. Therefore, the model developed in this work is able to simulate the real SP and WSP processes.

## 3. Results

### 3.1. Water Film

**Figure 7:**
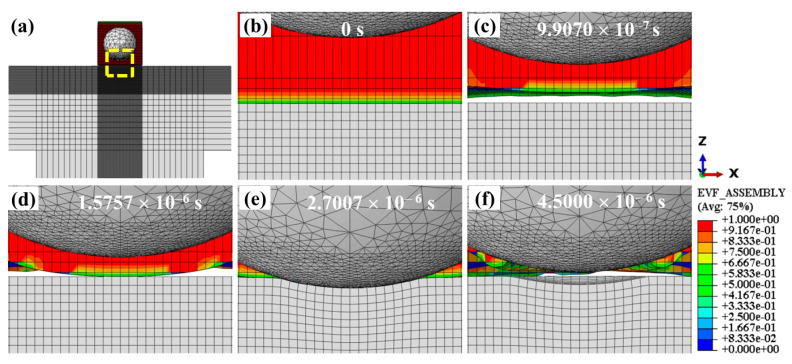
Water behavior of WSP-50 m/s at different times: (**a**) panoramic view of the model, (**b**–**f**) enlarged views of the yellow-boxed area in (**a**). A value of 0 (blue) indicates no water, while 1 (red) indicates the element is completely filled with water. Other colors represent partial filling.

The water behavior of WSP-50 m/s is shown in Figure 7. Figure 7b–f are the enlarged views of the yellow dashed box in Figure 7a. The colorful part is water, and the gray parts are shot and target. At first, the mixture of shot and water runs to the target and touches its upper surface (Figure 7b). Later, as shown in Figure 7c, the water bounces back and separates from the upper surface. Then, the water is pushed by the shot, and impacts the upper surface again (Figure 7d). After that, as seen in Figure 7e, the shot strikes the upper surface and creates a dimple. Finally, the mixture of shot and water leaves the upper surface (Figure 7f). White gaps between water and surface in Figure 7c,d and Figure 7f indicate transient void zones caused by water rebound and flow separation during high-velocity impact. These voids occur because the single-shot WSP model uses a limited water supply, a condition that may not arise in actual WSP processes.

**Figure 8:**
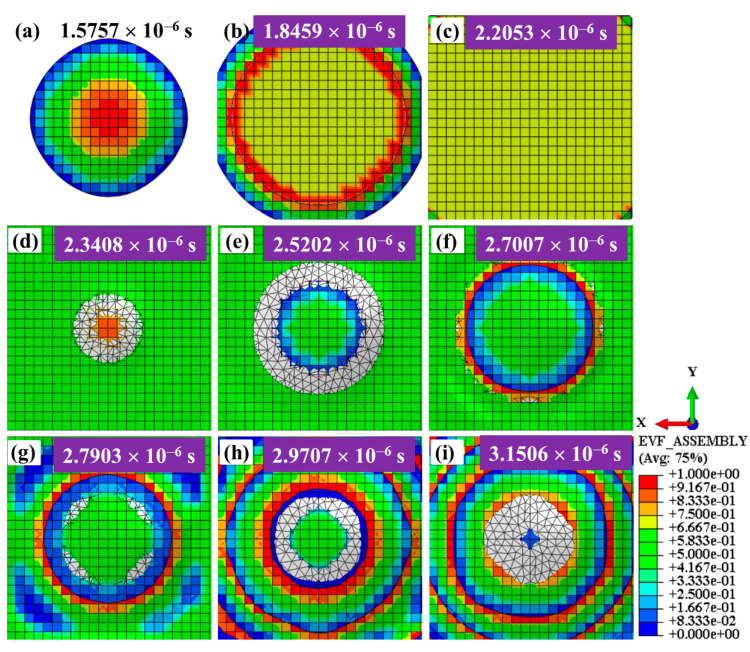
EVF evolution of the Eulerian meshes between the shot and the target at different stages: the shot approaches (**a**–**c**), impacts (**d**–**f**), and then departs from the target material (**g**–**i**).

EVF evolutions of Eulerian meshes between the shot and the target are studied to search for the water film during WSP. Figure 8 shows the behavior of water between shot and target in three stages: approach (a–c), impact (d–f), and departure (g–i). It illustrates the EVF evolutions during the period of 1.5757 × 10^−6^~3.1506 × 10^−6^ s with an observation area of 0.22 × 0.22 mm^2^. At the time of 1.5757 × 10^−6^ s (Figure 8a), the water pushed by the shot hits the surface again with a circular contact area. Then, the circular contact area becomes larger (Figure 8b). Finally, the water covers the entire observation area (Figure 8c). Figure 8d–f show the EVF evolutions during the impact process between shot and target. The values of EVF are always not null, which proves the existence of water film. The size of water film increases with the increase in contact area between shot and target. Figure 8g–i show the EVF evolutions during the process of leaving.

**Figure 9:**
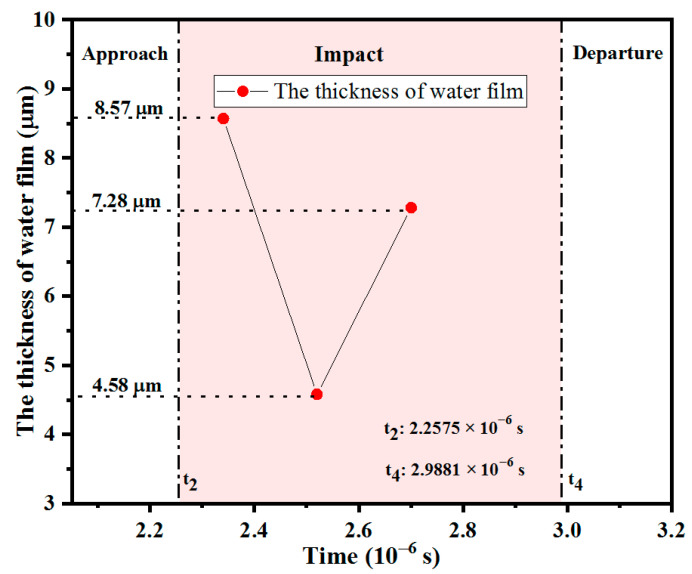
The thickness of the water film varies with time.

Since 0.6 mm (diameter of the shot) is much larger than 10 µm (mesh size), the surface of the shot is approximate to a flat surface within the area of a Eulerian element. Then, it is possible to calculate the thickness of water film (Figure 9). The thickness of water film changes with time and position. Water film thickness decreases as the impact contact area between the shot and target increases, and increases as the contact area decreases. At the time of 2.3408 × 10^−6^ s, 2.5202 × 10^−6^ s, and 2.7007 × 10^−6^ s, the thicknesses of the target center are 8.57 μm, 4.58 μm, and 7.28 μm, respectively.

### 3.2. Velocity

**Figure 10:**
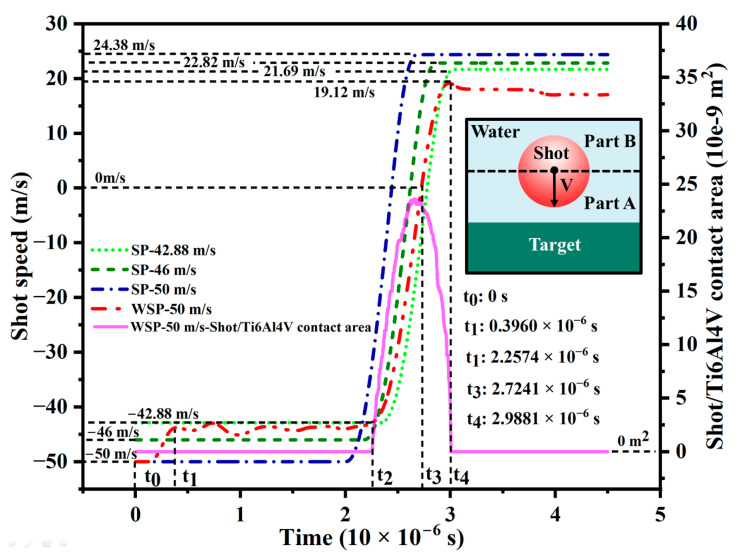
Shot velocity evolution and the contact area between shot and target.

The shot velocity evolutions of the four single-shot models are displayed in Figure 10. For convenient description, the water is separated into two parts (see the inset in Figure 10): the water between shot and target (named part A), and the remaining water (named part B). For WSP-50 m/s, the mixture of shot and water runs to the target with a velocity of 50 m/s at the beginning (time t_0_). Firstly, Part A reaches the upper surface of the target. Then, it is slowed down by the target. Simultaneously, there is a velocity difference between the shot and part A, which will hinder the shot’s movement. After that, the shot velocity decreases (t_0_~t_1_ period). Compared with part A, part B is affected slightly. At this time, the velocity of part B is higher than that of the shot, which will accelerate the shot. It explains why there is a modest increase after t_1_. Thereafter, the shot velocity fluctuates under the combined effect of obstruction from part A and acceleration from part B. The magnitude of velocity fluctuation depends on the resultant force of part A and part B. The data of contact area with time (magenta solid curve in Figure 10) is extracted to find the initial impact velocity between shot and target in the case of velocity fluctuation. It can be considered that the shot starts to hit the upper surface of the target when the value of the contact area is not null (time t_2_). Then, the 42.88 m/s effective impact velocity is revealed. After the collision between the shot and target, shot velocity drops rapidly to 0 m/s at the moment of t_3_. Then, it starts to accelerate in the opposite direction and separates from the target at the moment of t_4_. After separation, the shot velocity will be decayed to some extent due to the obstructive effect of water. For SP, the shot velocity evolution is much plainer. Before collision, the shot velocity maintains the initial velocity. Then, it decreases precipitously and accelerates in the opposite direction. After separation, the shot velocity will not change.

By comparing the velocity of the shot before and after contact with the target, the energy dissipated through material deformation can be calculated (Table 4). When the SP velocity increases from 42.88 m/s to 50 m/s, the dissipation rate increases from 74.41% to 76.18%. The WSP model exhibits the highest energy dissipation rate at 80.17%, indicating superior efficiency in facilitating material deformation.

### 3.3. Dimple

**Figure 11:**
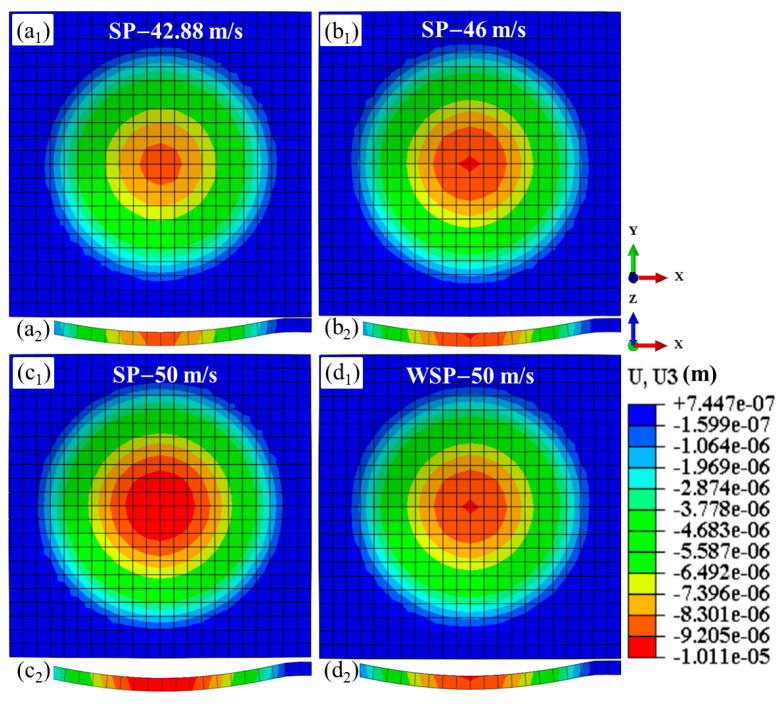
Surface topographies (**a_1_**–**d_1_**) and corresponding contours of dimples (**a_2_**–**d_2_**).

**Figure 12:**
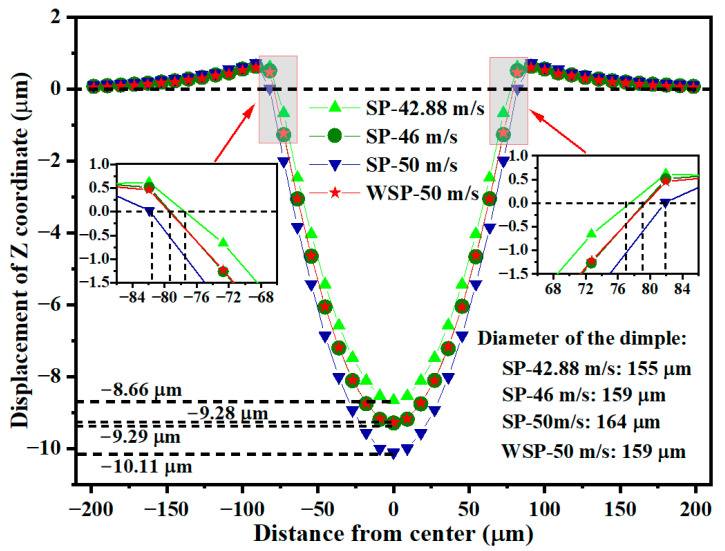
Specific data of the dimple sizes.

High-speed impact of shots on the target results in dimple formation and material extrusion around the dimple. This work focuses exclusively on counting and analyzing the dimple. The dimples created by four models are displayed in Figure 11. Figure 11a_1_–d_1_ are the surface topographies of SP-42.88 m/s, SP-46 m/s, SP-50 m/s, and WSP-50 m/s, respectively. Figure 11a_2_–d_2_ are the corresponding dimple contours past the dimple centers. The part where U3 is negative (inward concave) is the dimple. The location where U3 is null is the boundary of the dimple. It is easy to know that the dimples of SP-46 m/s and WSP-50 m/s are almost the same. The specific information of the dimples is visualized in Figure 12. With the SP velocity increases from 42.88 m/s to 46 m/s and 50 m/s, the dimple depth extends from 8.66 μm to 9.29 μm and 10.11 μm, and the dimple diameter raises from 154.99 μm to 158.52 μm and 163.64 μm. The depths and diameters of WSP-50 m/s and SP-46 m/s are similar, with the specific data of 9.28 μm, 158.87 μm and 9.29 μm, 158.52 μm, respectively. When compared to WSP-50 m/s with SP-46 m/s, the dimple depth is 0.01 µm shallower, while the dimple diameter is 0.35 µm larger. This means that WSP can obtain a larger diameter, even though the depth is shallower than that created by SP. WSP-50 m/s and SP-50 m/s have the same initial velocity, while the dimple depth and dimple diameter of WSP-50 m/s are 0.83 µm shallower and 4.77 µm smaller. So, treating WSP as SP is inaccurate, and ignores the water’s role.

### 3.4. Residual Stress

**Figure 13:**
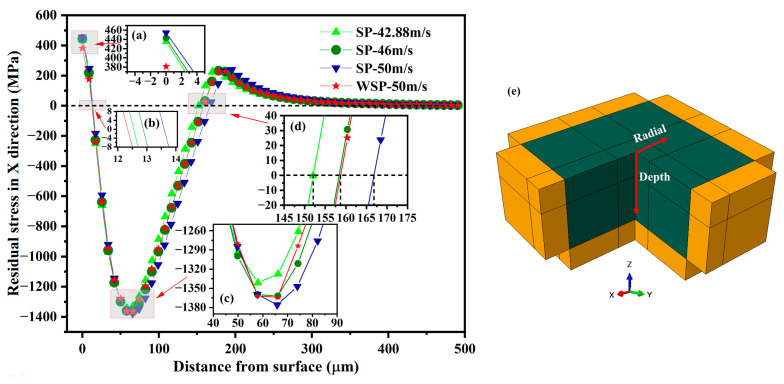
Residual stress distributions over depth (**a**–**d**); locations of the residual stress selected (**e**).

This paper analyzes the residual stress distributions in the depth direction and the radial direction of dimples. The residual stresses mentioned in the paper are stresses in the *x*-axis direction. The locations of the residual stress selected in the depth direction are shown in Figure 13e. All the residual stress curves are spoon-shaped (Figure 13). With the increase in depth, the residual stress shows a tensile stress state, a compressive stress state, and a tensile stress state, respectively. And finally, the residual stress tends to be zero. The specific values of residual surface stress (σ_RS1_), tensile stress layer depth (D_1_), maximum residual stress (σ_RS2_), maximum residual stress depth (D_2_), and compressive residual stress layer depth (D_3_) in the depth direction of the target center for the four models are shown in Table 5. When the velocity increases from 42.88 m/s to 46 m/s and 50 m/s, σ_RS1_, D_1_, σ_RS2_, D_2_, and D_3_ are all increased. Because WSP-50 m/s and SP-46 m/s have similar dimple sizes, comparing the residual stresses generated by the two models is essential and meaningful. When compared to WSP-50 m/s with SP-46 m/s, σ_RS1_ and D_1_ are 13.89% smaller and 4.19% thinner, respectively, while σ_RS2_, D_2_, and D_3_ are almost the same.

**Figure 14:**
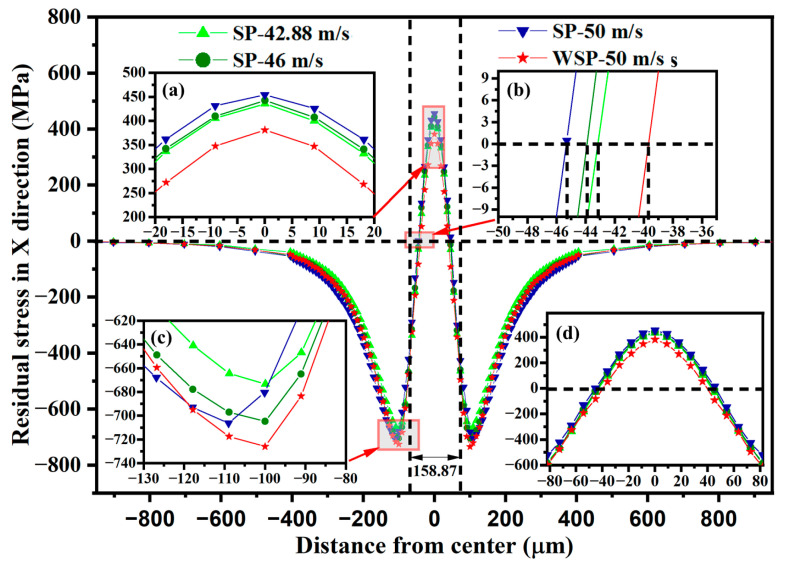
Residual stress distributions in the radial direction of dimples: (**a**–**c**) enlarged views of the area indicated by the corresponding red arrows, (**d**) enlarged view of the area between the two vertical dashed lines.

The residual stress distributions in the radial direction of four models are displayed in Figure 14. The locations of the residual stress selected in the radial direction on the upper surface of the target are shown in Figure 13e. The four models’ residual stress curves have a “W” shape, which are symmetrical about the dimple centers. The residual stress is in a tensile state near the dimple center. When the distance from the dimple center increases, the tensile residual stress decreases and changes to compressive residual stress. Then, the compressive residual stress continues to increase and reaches its maximum at the protrusion outside the dimple, and after that, the compressive residual stress starts to decrease to zero. Industrial application of the SP process usually needs to ensure that the SP coverage is greater than 100% (more than 98% actual coverage can be considered as 100%), so the residual stress distribution in the dimple area is meant for application. The residual stress curves between the two vertical dashed lines shown in Figure 14 are the residual stress distributions in the radial direction of dimples, which are enlarged in Figure 14d. The residual stress is in a tensile state near the dimple center, while it is in a compressive state around the dimple’s edge. In the dimple’s radial direction, the maximum tensile residual stress (σ_RS3_), the area of tensile residual stress (L_TRS_) (measured by the length of the tensile residual stress area), the maximum compressive residual stress (σ_RS4_), the area of compressive residual stress (L_CRS_) (measured by the length of the compressive residual stress area), and the percentage of compressive residual stress (P_CRS_) (measured by L_CRS_/(L_CRS_ + L_TRS_)) are listed in Table 6. Compared WSP-50 m/s with SP-46 m/s, σ_RS4_, L_CRS_, and P_CRS_ are 5.4% bigger, 12.43% larger, and 12.17% higher, while σ_RS3_ and L_TRS_ are both smaller with varying degrees. For evaluating the residual stress distribution, the standard deviation is solved for the residual stress. The standard deviations for the residual stresses of SP-42.88 m/s, SP-46 m/s, SP-50 m/s, and WSP-50 m/s are 304.56 MPa, 304.51 MPa, 298.48 MPa, and 286.59 MPa, respectively. The standard deviation of WSP is smaller than that of SP, which means the residual stress distribution of WSP is more uniform than that of SP.

### 3.5. Effect of Velocity During WSP

**Figure 15:**
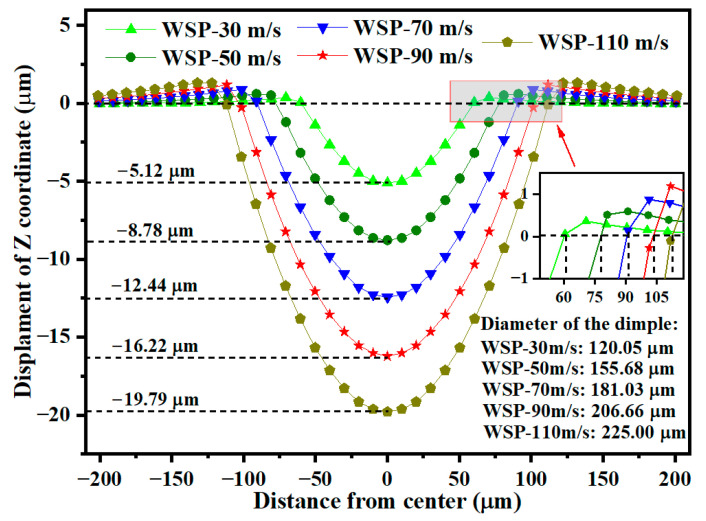
Specific data of the dimple sizes for different velocities.

Previous research has extensively examined the effects of SP parameters [13,14,15,16,17,18]. This work focuses specifically on the influence of velocity (Section 3.5) and liquid viscosity (Section 3.6) on dimple sizes and residual stresses during WSP. WSP, like conventional SP, can be performed at velocities of up to 100 m/s [32]. In this work, five WSP velocities were investigated: 30 m/s (WSP-30 m/s), 50 m/s (WSP-50 m/s), 70 m/s (WSP-70 m/s), 90 m/s (WSP-90 m/s), and 110 m/s (WSP-110 m/s). The models, based on Figure 3b, utilized hemispherical shots. All parameters were held constant except for velocity. For the 50 m/s condition, both complete sphere and hemisphere models were compared. The resulting dimple diameters were 163.64 μm and 155.68 μm, respectively, with a difference of 4.86%. The maximum residual compressive stresses were 1364.13 MPa and 1267.83 MPa, respectively, with a difference of 7.06%. Both models produced similar numerical results and demonstrated consistent trends. Due to its lower computational cost, the hemispherical model was selected for further analysis of velocity parameters. As velocity increased from 30 m/s to 110 m/s, dimple depth increased from 5.12 μm to 19.79 μm, and dimple diameter increased from 120.05 μm to 225.00 μm (Figure 15).

**Figure 16:**
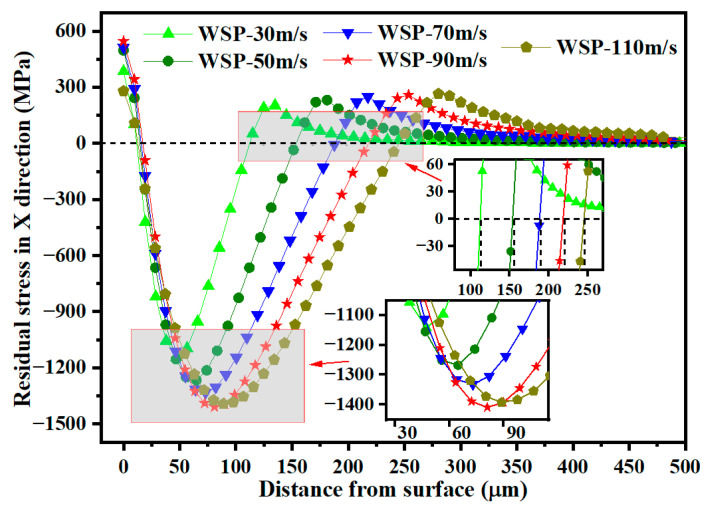
Residual stress distributions over the depth for different velocities.

Figure 16 shows the residual stress distribution along the depth direction at the dimple center. Increasing shot velocity raises kinetic energy, which intensifies material deformation and deepens the deformation layer. This deeper deformation layer results in both a greater D_2_ and an increased D_3_. As WSP velocity increases from 30 m/s to 90 m/s, the σ_RS2_ rises by 267.04 MPa, from 1142.22 MPa to 1409.26 MPa. The residual stress in a specific region is determined by the deformation gradient in adjacent regions. When shot velocity reaches 110 m/s, the *σ*_RS2_ decreases compared to 90 m/s, suggesting that the deformation gradient at WSP-110 m/s is less favorable. Stress relaxation may be attributed to the dislocation gliding after reaching a critical dislocation density value [26].

**Figure 17:**
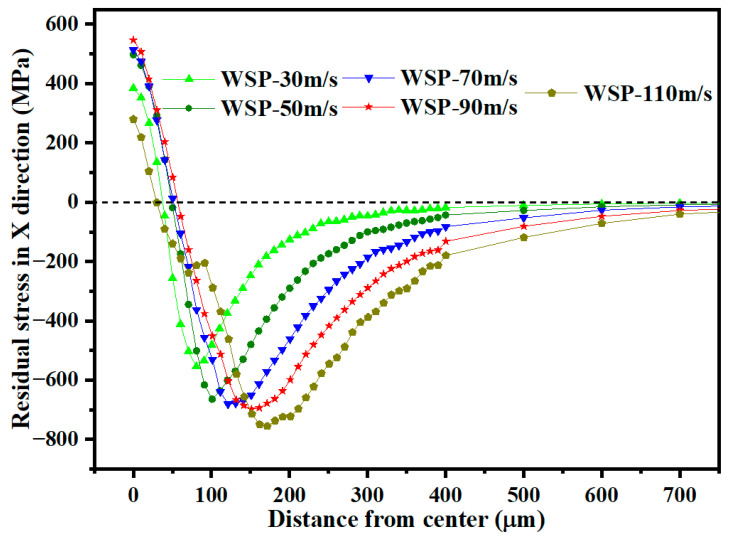
Residual stress distributions in the radial direction of dimples for different velocities.

Figure 17 presents the residual stress distribution in the radial direction. As velocity increases, *σ*_RS2_ gradually shifts away from the dimple center. At a velocity of 110 m/s, the residual stress distribution in the surface layer deviates from the expected pattern. This deviation is likely attributable to insufficient material flow caused by the high shot velocity.

When the velocity increases from 30 m/s to 110 m/s, dimple depth, dimple diameter, D_2_, and D_3_ increase. The *σ*_RS2_ in the depth direction was observed at a velocity of 90 m/s. At 110 m/s, the radial residual stress distribution exhibited abnormal characteristics. Based on residual stress analysis, the optimal velocity is 90 m/s.

### 3.6. Effect of Viscosity During WSP

**Figure 18:**
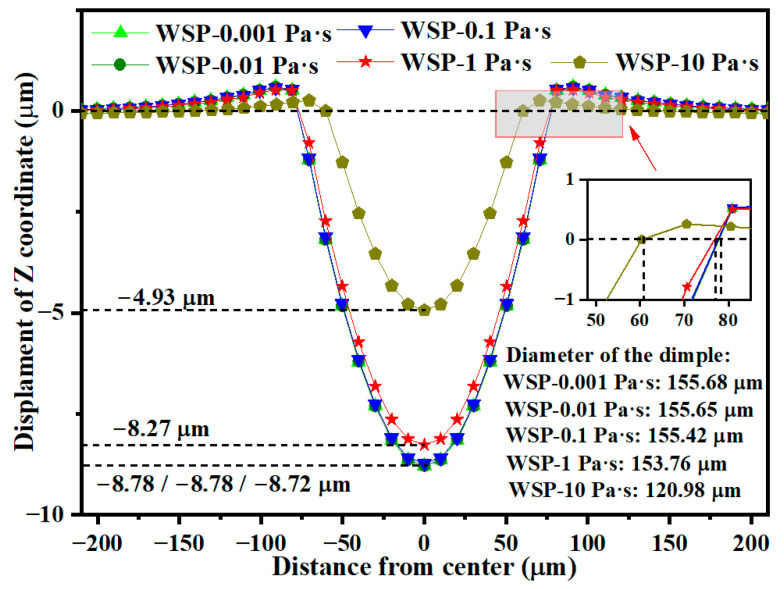
Specific data of the dimple sizes for five different viscosities.

During WSP, the high-velocity mixture of shot and liquid impacts the target. The influence of liquid viscosity on WSP performance requires a detailed investigation. Liquids with excessively high viscosity exhibit poor fluidity and are unsuitable for the wet blasting process. This study examines five viscosities: 0.001 Pa·s (WSP-0.001 Pa·s), 0.01 Pa·s (WSP-0.01 Pa·s), 0.1 Pa·s (WSP-0.1 Pa·s), 1 Pa·s (WSP-1 Pa·s), and 10 Pa·s (WSP-10 Pa·s). All models maintain identical parameters except for viscosity and utilize the same hemispherical geometry described in Section 3.5. Figure 18 presents the effect of liquid viscosity on the dimple size of the target. Dimple size is almost unchanged when the viscosity ranges from 0.001 to 0.1 Pa·s. Increasing viscosity from 0.1 Pa·s to 1 Pa·s results in minor changes: dimple depth decreases by 5.16% and dimple diameter decreases by 1.07%. When viscosity increases from 1 Pa·s to 10 Pa·s, dimple size changes substantially, with crater depth decreasing by 40.38% and crater diameter decreasing by 21.32%.

**Figure 19:**
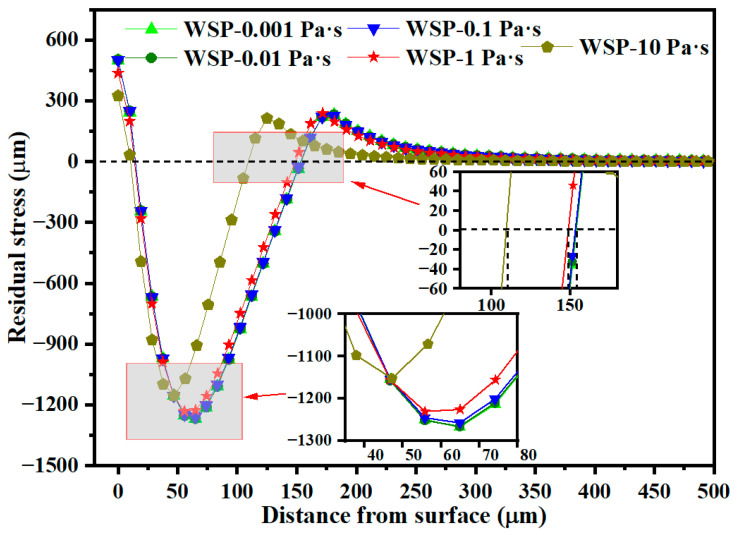
Residual stress distributions over depth for different viscosities.

Figure 19 reveals the residual stress distribution along the depth direction at the crater center. The residual stress remains largely unchanged as viscosity varies from 0.001 to 0.1 Pa·s, consistent with the variation in dimple size. When liquid viscosity increases from 0.1 to 1 Pa·s, the σ_RS2_ decreases by 2.16 percent, from 1258.19 MPa to 1231.04 MPa. Further increasing viscosity from 1 to 10 Pa·s results in a 6.36 percent reduction in σ_RS2_, from 1231.04 MPa to 1152.71 MPa, and a 26.25 percent decrease in D_3_, from 148.73 μm to 109.32 μm.

**Figure 20:**
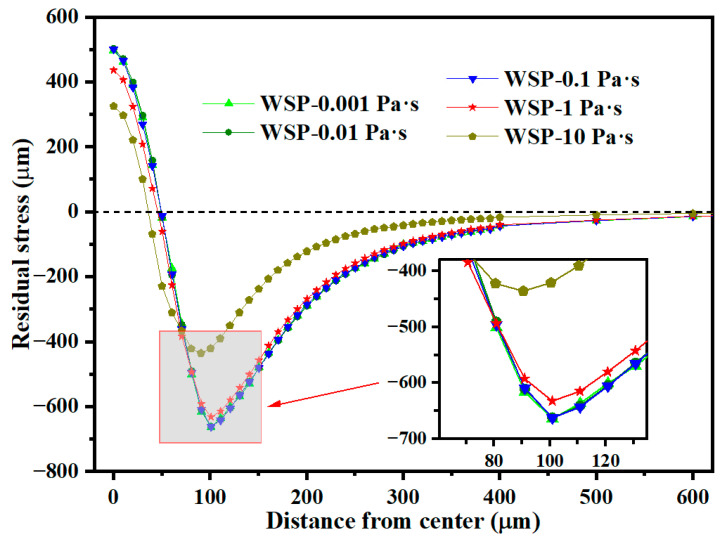
Residual stress distributions in the radial direction of dimples for different viscosities.

Figure 20 shows the residual stress distribution in the radial direction. Residual stress remains largely unchanged as viscosity increases from 0.001 to 0.1 Pa·s. When viscosity rises from 0.1 to 1 Pa·s, the σ_RS2_ decreases by 4.56% from 662.37 MPa to 632.18 MPa. A further increase in viscosity from 1 to 10 Pa·s results in a 31.11% reduction in σ_RS2_, from 632.18 MPa to 435.47 MPa.

Overall, the dimple size and residual stress distribution remain relatively stable as the viscosity increases from 0.001 to 0.1 Pa·s. When viscosity ranges from 0.1 to 10 Pa·s, both dimple size and sRS2 decrease as viscosity increases. The reduction in dimple size and sRS2 is more pronounced between 1 and 10 Pa·s than that between 0.1 and 1 Pa·s. In engineering applications, a decrease in sRS2 is generally undesirable, whereas a reduction in dimple size is advantageous. The optimal liquid viscosity parameter should be selected based on whether surface roughness or residual compressive stress is the primary concern in a given application.

## 4. Discussion

### 4.1. Water Film Formation

**Figure 21:**
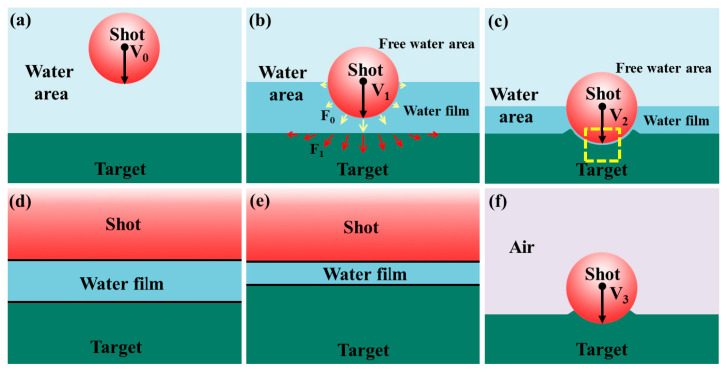
Schematic diagram illustrating water film formation during WSP and shot impact during SP: (**a**) The shot and water mixture approaches the target. (**b**,**c**) A water film forms as the shot nears the target, with thickness varying by position. (**d**,**e**) Enlarged views of the yellow-dashed area in subfigure (**c**). (**f**) Schematic of shot impact during SP for comparison with WSP.

The schematic diagram of water film formation is drawn in Figure 21 based on the above results and analysis. Firstly, the mixture of shot and water rushes to the target surface at a particular velocity of V_0_ (Figure 21a). Then, as the mixture approaches the target surface, the shot velocity drops to V_1_ due to the obstructive effect of the target. At this point, the water can be divided into two parts: the water film between the shot and the target (also named part A in Section 3.2), and the free water area (also named part B in Section 3.2). The water film not only impacts the target by its kinetic energy but also transfers the energy from the shot to the target (Figure 21b). After that, the shot hits the target surface with a co-product of a dimple. Meanwhile, the water film thickness changes to microscale (Figure 21c). Figure 21d,e are the enlarged views of the yellow dashed box in Figure 21c. The water film thickness changes with time and position (Figure 21d,e). Figure 21f is the schematic diagram of the dimple created by SP.

### 4.2. The Effect of Water Film

**Figure 22:**
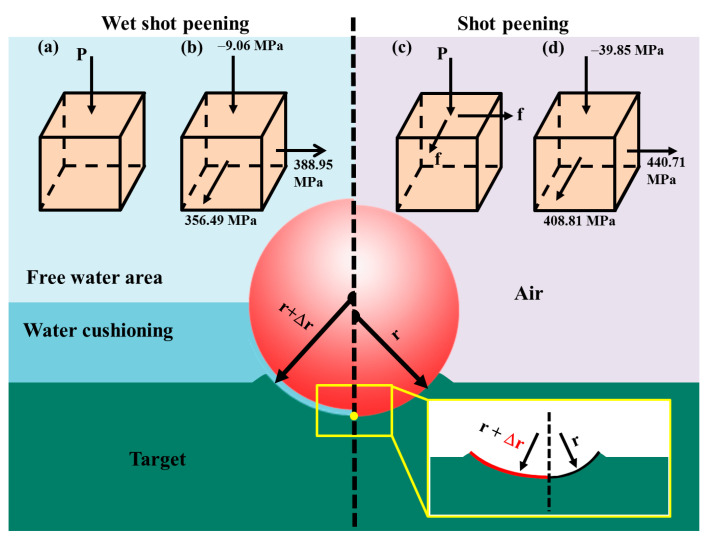
The effect of water film on the dimple and residual stress: schematic diagram of forces acting on the center of the target during WSP and SP (**a**,**c**), and the residual stresses after impact (**b**,**d**).

The presence of a water film decreases the surface roughness. WSP-50 m/s can form a dimple with a bigger diameter than SP-46 m/s, despite the fact that the dimple depth is shallower (Section 3.3). The only difference between the two processes is the water medium. The water film during WSP is equivalent to a layer of cladding with thickness Δr on the shot of radius r. When the dimple depths of WSP and SP are comparable, the dimple diameter created by WSP is larger (Figure 22). Conversely, when producing dimples with a similar diameter, WSP generates shallower depths, resulting in lower surface roughness. Feldmann attributed this to the buffering effect of the water film [26].

The water film increases lubrication and reduces friction, which results in a more favorable stress distribution. Compared with SP, the friction between water and the target surface can be ignored in WSP. The forces during processing at the target center (yellow point in Figure 22) of WSP and SP are revealed in Figure 22a,c. The final forces after processing at the target center are shown in Figure 22b,d. The lower friction coefficient results in lower σ_RS3_ and higher σ_RS4_, which is advantageous to the improvement of fatigue life. The frictional force is much smaller than the normal impact force and has little influence on the inner stress, which explains why the stress distributions of WSP and SP are similar except for those in the targets’ surface layer. The presence of the water film reduces the standard deviation of residual stresses across the target material surface, thereby promoting a more uniform stress distribution.

The water film affects the impact energy conversion rate. When there is friction, an additional load is required for producing a given size dimple. The extra load can be up to 5% total load [41]. SP-46 m/s and WSP-50 m/s produced similarly sized dimples. However, SP-46 m/s required approximately 15% more impact energy than WSP-50 m/s (the effective impact velocity WSP-50 m/s is 42.88 m/s, see Figure 10). This is because SP differs from WSP not only in terms of friction coefficient but also in the impact energy conversion rate. As shown in Table 4, the WSP-50 m/s has the highest energy dissipation rate at 80.17%, indicating superior efficiency in facilitating material deformation.

In conclusion, the water film between shot and target can play the roles of buffer and lubrication, which will have effects on shot velocity, dimple size, residual stress distribution, and impact energy conversion rate. These may be the intrinsic reasons for the difference in surface integrity between WSP and SP. The water film helps to increase the dimple diameter, raise σ_RS4_, elevate P_CRS_, and acquire a more equally distributed residual stress in the surface layer. The larger curvature radius of the dimple can lower stress concentration, and the higher compressive residual stress can inhibit crack propagation, which is more beneficial for improving fatigue life.

## 5. Conclusions

A detailed study of WSP using the FEM is conducted in this work, and the following results are obtained.

1. This work provides the first comprehensive modeling of the water-film effect using the CEL method during WSP. The water film thickness is at a microscale under the following conditions: the shot is made of ceramic with a diameter of 0.6 mm, the target is made of Ti6Al4V, and the initial velocity is 50 m/s.
2. The water film during WSP is comparable to a layer of cladding with a thickness Δr on the shot surface. When producing a dimple with a given depth, the bigger shot will create a larger-diameter dimple.
3. Water film induced by WSP affects the residual stress distribution in the target’s surface layer. WSP can obtain lower σ_RS1_ and thinner D_1_ in the depth direction. Compared with σ_RS1_ and D_1_ of SP-46 m/s, those of WSP-50 m/s are 13.89% smaller and 4.19% thinner, respectively. In the radial direction of the dimple, WSP can form higher σ_RS4_, larger L_CRS_, and more equally distributed residual stress. The σ_RS4_ and P_CRS_ of WSP-50 m/s are 5.4% greater and 12.17% larger than those of SP-46 m/s, respectively. The standard deviation for the residual stress of WSP-50 m/s is 17.92 MPa smaller than that of SP-50 m/s, which means the residual stress distribution of WSP is more uniform than that of SP.
4. The σ_RS2_ increases as the WSP velocity rises from 30 m/s to 90 m/s. At 110 m/s, the σ_RS2_ decreases relative to 90 m/s. Therefore, 90 m/s is identified as the optimal velocity parameter for maximizing residual compressive stress.
5. Dimple size and residual stress distribution remain largely unchanged when viscosity ranges from 0.001 to 0.1 Pa·s. For viscosities between 0.1 and 10 Pa·s, both dimple size and σ_RS2_ decrease as viscosity increases. The optimal liquid viscosity parameter should be selected based on whether surface roughness or residual compressive stress is the primary consideration in the specific application.

## Figures and Tables

**Table 1:** Properties of Ti6Al4V and ceramic.

Material	Elasticity Modulus (E, Pa)	Density (kg/m^3^)	Poisson Ratio
Ti6Al4V	1.214 × 10^11^	4510	0.30
Ceramic	3.500 × 10^11^	3800	0.26

**Table 2:** Parameters of Johnson–Cook constitutive law for Ti6Al4V.

Material	A (Pa)	B (Pa)	C	*n*	m	T_melt_ (K)	T_room_ (K)	$\overset{•}{\varepsilon_{0}}$ (1/s)
Ti6Al4V	1.098 × 10^9^	1.092 × 10^9^	0.014	0.93	1.1	1878	298	1

**Table 3:** Parameters of Mie–Grüneisen equations of state for water.

Density (kg/m^3^)	Viscosity (Pa·s)	*c*_0_ (m/s)	s	Γ_0_
983	0.001	1435	0	0

**Table 4:** Energy of different models.

Model	Energy (Before Impact, J)	Energy (After Impact, J)	Dissipation (J)	Dissipation Rate (%)
SP-42.88 m/s	4.69 × 10^−4^	1.20 × 10^−4^	3.49 × 10^−4^	74.41
SP-46 m/s	5.40 × 10^−4^	1.33 × 10^−4^	4.07 × 10^−4^	75.37
SP-50 m/s	6.38 × 10^−4^	1.52 × 10^−4^	4.86 × 10^−4^	76.18
WSP-50 m/s	4.69 × 10^−4^	0.93 × 10^−4^	3.76 × 10^−4^	80.17

**Table 5:** Specific data of residual stress over depth.

Model	σ_RS1_ (MPa)	D_1_ (μm)	σ_RS2_ (MPa)	D_2_ (μm)	D_3_ (μm)
SP-42.88 m/s	435.90	12.55	−1341.40	58.01	152.07
SP-46 m/s	442.90	12.88	−1362.06	61.97	158.37
SP-50 m/s	454.47	13.60	−1375.85	65.82	166.83
WSP-50 m/s	381.40	12.34	−1364.13	61.94	158.72
Difference	−13.89%	−4.19%	0.15%	−0.05%	0.22%

Note: “Difference” means (X_WSP-50m/s_ − X_S-46m/s_)/X_SP-46m/s_, X is the corresponding data (σ_RS1_, D_1_, σ_RS2_, D_2_, D_3_).

**Table 6:** Specific data of residual stress in the radial direction of dimples.

Model	σ_RS3_ (MPa)	L_TRS_ (μm)	σ_RS4_ (MPa)	L_CRS_ (μm)	P_CRS_ (%)
SP-42.88 m/s	435.90	86.35	−524.19	68.64	44.29
SP-46 m/s	442.90	87.82	−552.81	70.70	44.60
SP-50 m/s	454.47	90.68	−521.13	72.96	44.59
WSP-50 m/s	381.40	79.38	−582.48	79.49	50.03
Difference	−13.89%	−9.61%	5.37%	12.43%	12.17%

Note: “Difference” means (X_WSP-50m/s_ − X_S-46m/s_)/X_SP-46m/s_, X is the corresponding data (σ_RS3_, L_TRS_, σ_RS4_, L_CRS_, P_CRS_).

## Data Availability

The original contributions presented in this study are included in the article. Further inquiries can be directed to the corresponding author.
